# Progress in Diseases of the Heart

**Published:** 1900-10-13

**Authors:** 


### Progress in Diseases of the Heart.

**Experiment.**—Cushny and Matthewson,[1] experimenting on the mammalian heart by means of simple electric shocks, find that there exists a refractory period during which stimulation is without effect. For the ventricle it extends from a time just before contraction commences to a time just anterior to the completion of the systole. The artificially-produced contraction differs in no way from the natural. They state : "The whole of the phenomena of the forced contraction of the ventricle are thus merely modifications of those occurring in the natural cycle, and every gradation between the completely normal and the most marked deviation from it may be elicited by altering the length of the interval between the spontaneous and forced contraction. The importance of this fact in explaining irregularities of the heart's action cannot be overestimated."

This phenomenon was also noticed in the auricle.

In delirium cordis he states that the clinical sphygmogram resembles that obtained from dogs when the auricle is undergoing fibrillary contractions. Section of the vagi dissipates these, and Matthews and Cash have noted that atropine will prevent the irregularity and final delirium of the heart from aconitine. "It would be of interest to ascertain the effect of atropine upon clinical delirium cordis."

**Therapeutics.**—Thomson[2] states that conditions of acute parenchymatous degeneration of the heart muscle, whether due to diphtheritic or other toxins, are injured by the administration of digitalis; for, the muscle wall being impaired, the fibres have lost much of their contractility. Rest is the great essential in all cases of carditis. Aconite, pushed to producing its specific effects, will often improve marvellously the whole clinical aspect of a case of carditis. It relieves cardiac pain and slows the heart beat. Belladonna is often beneficial in mitral stenosis in that it allays spasm and restores the normal rhythm to disturbed conditions of rhythmically acting muscular fibre. His experience is that myocardial degeneration is one of the commonest and most efficient of all causes of failure of compensation in the heart diseases of patients after middle life, and that it precedes valvular incompetence in a large proportion of them. Thus, a man may suffer from dyspnœa, flatulence, disturbed sleep, and show a very feebly-acting heart without disease; death may occur suddenly, and at the necropsy atheroma of the arteries is found. In such a case general health is the first consideration, and digitalis is of inestimable service. Nitroglycerine should be given with each dose to counteract the arterial constriction which digitalis causes. Drugs, however, can never be other than temporary makeshifts, and in a progressive degenerative process they are powerless to cure it. Fresh air is of the utmost importance, and drugs such as iron are therefore of great importance in aiding the oxygen-carrying functions of the blood. Soulier[3] claims that just as in the case of tonic contractions of the extensors during flexion of a limb, so in the heart there is no perfect systole without the opposition of a synchronous elasticity. This elasticity is diastolic, and is caused by the force of the auricular systole. The heart therefore has a propelling and aspirating force. This elasticity in diastole is increased by digitalis, strychnine and caffeine, but diminished by atropine. The author showed tracings from a dog under the influence of digitalis, where this elasticity was apparent owing to the slowing of the heart. Digitalis is contra-indicated in aortic insufficiency, but may be useful in pneumonia on account of the increase it produces in the aspiratory force. Heinrich Stern[4] extols the glucoside "Adonidin" as a very good substitute for digitalis, or in preference to that drug. He has used it frequently, and has not found that it has a cumulative action; it is well borne where digitalis causes toxic symptoms; it is more prompt and energetic in its action, and in spite of this can be given with safety in such pathological conditions as "fatty degeneration of the heart, pericarditis, simple hypertrophy and certain atheromatous conditions." "In rapidity of action it almost equals nitroglycerine;" it is more certain than caffeine, sparteine sulphate, convallamarin, strophanthus, digitalis, or its glucosides. And it is more permanent in its effects than any of these or nitroglycerine. It has little diuretic action in health, but in "certain affections of the kidney and pyretic conditions" it acts in a more decided manner. Stern is in the habit of giving the drug in quantities upwards of ·002 gm. (·031 gr.) to ·01 gm. (·154 gr.) three times daily, in pill, powder or hypodermically, either simply or in combination. He quotes twelve cases where its use was attended with markedly beneficial results, comprising circulatory disturbances in nephritis, angina pectoris and others.

Porter[5] holds that the cumulative action of digitalis is due to the contraction of the arterioles, and the shutting off of nutrition; that the drug is of use only in mitral lesions and should be discontinued as soon as these have been overcome. Potain[6] considers digitalis to be contra-indicated when there does not exist inequality, irregularity, and insufficiency of the cardiac pulsation in myocardial lesions, aortic incompetence (unless there be undue acceleration of the beats), dyspepsia and a cachectic condition. He does not consider a permanently infrequent pulse as a contra-indication. The bad results of digitalis are first of all nocturnal delirium, pallor, coldness of the extremities, trembling and contraction of the pupil. Death from digitalis is most frequently met with in Bright's disease, arthritic and anæmic subjects, and in persons with aortic incompetence or delirium tremens. Idiosyncrasies may be met with, such as melancholia, night terrors, and even pulmonary apoplexy. Sidney Martin,[7] speaking of the use of digitalis in heart disease, states that it is contra-indicated in advanced aortic regurgitation and obstruction. In early obstruction small doses may be beneficial. In combined mitral and aortic disease the chief indication is from the pulse, for though there may be typical physical signs of aortic regurgitation, yet there may be associated with that the very frequent pulse of mitral disease, or there may be less frequency but irregularity. In these cases small doses do good. In many cases of mitral disease ensuing on aortic regurgitation, digitalis does harm by increasing the dilatation of the ventricle, and stimulating remedies such as strychnia are better.

Schott[8] reviews the treatment of "fatty heart," by which is indicated an overgrowth or intermixture of fat as distinct from fatty degeneration.

After the ancient treatment by bleeding, and later by purgation, had fallen into proper disuse, the salts of iodine were strongly recommended. These he holds to be injurious to the general nutrition, and are more likely to reduce the general obesity than that of the heart. Similarly, thyroidin may prove unsatisfactory and even dangerous. Of oöphorin tabloids he can state nothing at present. With the older mineral waters of Marienbad, Karlsbad, Tarasp, &c., the diminution in weight may be so rapid as materially to affect the strength, with general debility, chlorosis, anæmia and other troubles. And such waters as those of Kissingen, Kreuznach, Nauheim, &c., have been used in too great quantities, with the result of gastric catarrh and obstinate constipation, and no guarantee against recurrence. The Harvey-Banting treatment aimed at limiting the carbohydrate and fatty ingesta: this was attended by a dangerous loss of albuminates to the system. Oertel's hill-climbing and restricted fluid resulted often in dyspnœa, dyspepsia, insomnia, and even nephritis. In general it is forgotten that a fatty heart is one with muscular insufficiency, and we cannot limit the albuminates of the body without limiting those of the heart in some degree, and thus weakening it further. Dietetic measures must be conjoined with the mechanical measures first introduced by Stokes. Mistakes were made at first in making the exercises too great (Oertel), but in exercises with resistance of various strengths, as recommended by August Schott, without excess and without rest after meals, there is great advantage.

Baths are now added to the treatment, of gradually increasing concentrations of salt, and carbonic acid effervescent baths of moderate temperature. The effervescent baths seem to benefit anæmia and chlorotic patients especially.

The musculature, he continues, is improved with the loss of fat; but this loss must never be too rapid, else debility and dyspnœa from weakened heart muscle will ensue. He also gives tracings from cases before and after treatment.

Mayer[9] gives the quantities of salts required to produce artificial Nauheim baths. He uses a mixture of

| | |
|---|---|
| Sod. chloride ... ... | 30 lbs. (sea salt) |
| Pot. chloride ... ... | 10 ozs. |
| Cal. chloride ... ... | 20 ozs. |
| Mag. chloride ... ... | 8 ozs. |

Of this he uses 3 lbs. in 30 gallons water, and increases gradually the strength to 5 lbs. in 30 gallons. He produces the effervescent carbonic acid bath by using ¾ lb. HCl to ½ lb. bicarb. sod. for the mild bath, double these quantities for the medium, and for the strongest 3 lbs. of acid on 2 lbs. of salt.

He recommends the acid to be put into the bath by means of a bottle, which is unstoppered at the bottom of the water, and thus the acid is distributed over the lower surface. Or six to eight 2-oz. cakes of acid sod. sulphate may be used with bicarbonate of soda. The body is totally immersed, with the head supported on webbing stretched across the bath. The acid cakes are placed on tinfoil in the bath, and so do no harm to the material of the bath.

He holds that the artificial method has just as good results as the actual baths at Nauheim, provided the method of procedure is the same.

[1] Med. Chron., Feb. [2] Med. Rec., Mar. 17. [3] Med. Chron., April. [4] Merck's Archives, May. [5] Med. Rec., May 12. [6] Brit. Med. Jour., May 12. [7] Clin. Jour., June 20. [8] Med. Rec., Mar. 24. [9] Med. Rec., May 26.

